# A conserved MADS-box phosphorylation motif regulates differentiation and mitochondrial function in skeletal, cardiac, and smooth muscle cells

**DOI:** 10.1038/cddis.2015.306

**Published:** 2015-10-29

**Authors:** W Mughal, L Nguyen, S Pustylnik, S C da Silva Rosa, S Piotrowski, D Chapman, M Du, N S Alli, J Grigull, A J Halayko, M Aliani, M K Topham, R M Epand, G M Hatch, T J Pereira, S Kereliuk, J C McDermott, C Rampitsch, V W Dolinsky, J W Gordon

**Affiliations:** 1 Department of Human Anatomy and Cell Science, University of Manitoba, Winnipeg, MB, Canada; 2 The Diabetes Research Envisioned and Accomplished in Manitoba (DREAM) Theme of the Children's Hospital Research Institute of Manitoba, University of Manitoba, Winnipeg, MB, Canada; 3 Department of Biology, York University, Toronto, ON, Canada; 4 College of Nursing, University of Manitoba, Winnipeg, MB, Canada; 5 Department of Mathematics and Statistics, York University, Toronto, ON, Canada; 6 Department of Physiology and Pathophysiology, University of Manitoba, Winnipeg, MB, Canada; 7 The Biology of Breathing Theme of the Children's Hospital Research Institute of Manitoba, University of Manitoba, Winnipeg, MB, Canada; 8 Department of Human Nutritional Sciences, University of Manitoba, Winnipeg, MB, Canada; 9 Huntsman Cancer Institute, University of Utah, Salt Lake City, UT, USA; 10 Department of Biochemistry and Biomedical Sciences, McMaster University, Hamilton, ON, Canada; 11 Department of Pharmacology and Therapeutics, University of Manitoba, Winnipeg, MB, Canada; 12 Agriculture and Agrifood Canada, Morden, MB, Canada

## Abstract

Exposure to metabolic disease during fetal development alters cellular differentiation and perturbs metabolic homeostasis, but the underlying molecular regulators of this phenomenon in muscle cells are not completely understood. To address this, we undertook a computational approach to identify cooperating partners of the myocyte enhancer factor-2 (MEF2) family of transcription factors, known regulators of muscle differentiation and metabolic function. We demonstrate that MEF2 and the serum response factor (SRF) collaboratively regulate the expression of numerous muscle-specific genes, including microRNA-133a (miR-133a). Using tandem mass spectrometry techniques, we identify a conserved phosphorylation motif within the MEF2 and SRF *M*cm1 *A*gamous *D*eficiens *S*RF (MADS)-box that regulates miR-133a expression and mitochondrial function in response to a lipotoxic signal. Furthermore, reconstitution of MEF2 function by expression of a neutralizing mutation in this identified phosphorylation motif restores miR-133a expression and mitochondrial membrane potential during lipotoxicity. Mechanistically, we demonstrate that miR-133a regulates mitochondrial function through translational inhibition of a mitophagy and cell death modulating protein, called Nix. Finally, we show that rodents exposed to gestational diabetes during fetal development display muscle diacylglycerol accumulation, concurrent with insulin resistance, reduced miR-133a, and elevated Nix expression, as young adult rats. Given the diverse roles of miR-133a and Nix in regulating mitochondrial function, and proliferation in certain cancers, dysregulation of this genetic pathway may have broad implications involving insulin resistance, cardiovascular disease, and cancer biology.

Extensive cell and molecular analysis has identified numerous extracellular cues that regulate skeletal, cardiac, and smooth muscle myogenesis.^1, 2, 3^ However, the impact of nutrient availability and metabolic excess on myogenesis has been less studied. One of the most important periods of myogenesis is during gestation, and fetal exposure to metabolic diseases dramatically alters the development and post-natal metabolism of muscle. For example, the offspring of overweight pregnant rats have reduced muscle fibers and nuclei.^4^ In addition, fetal exposure to diabetes during pregnancy increases the risk for early-onset insulin resistance in the offspring;^5^ however, the key molecular regulators responsible for fetal metabolic programming have not been characterized in muscle tissues.

During mammalian development, myogenic precursors derived mostly from mesenchymal populations commit to one of three main muscle lineages: skeletal, cardiac, and smooth muscles.^2, 6, 7, 8^ Among the core muscle transcriptional regulators is the myocyte enhancer factor-2 (MEF2) family of transcription factors, where gene-targeting studies in both *Drosophila* and mammals have reported essential roles for the MEF2 family in the development and post-natal remodeling of all muscle lineages.^9^

The MEF2 family is composed of four transcription factors, MEF2-A to -D, which have both overlapping and non-redundant functions. The amino terminus of MEF2 proteins contains a highly conserved 58-amino acid *M*cm1 *A*gamous *D*eficiens *S*RF (MADS)-box that mediates dimerization and binding to a cognate *cis* element (T/C)TA(A/T)_4_TA(G/A).^10^ The transcriptional activity of MEF2 proteins, along with their ability to bind DNA, is highly regulated by post-translational modification, including phosphorylation.^11, 12, 13^ In mammalian cells, the only other MADS-box containing transcription factor is the serum response factor (SRF), which binds to a similar cognate *cis* element, CC(A/T)_6_GG,^14^ and has also been implicated in smooth muscle and striated muscle differentiation.^3, 15, 16^ Given their similar structure and overlapping function, surprisingly little is known regarding the cooperation between MEF2 and SRF proteins during muscle differentiation, and whether these MADS-box factors serve to coordinate aspects of mitochondrial function.

MEF2 proteins regulate metabolism and muscle fiber-type by direct transcriptional activation of numerous enzymes and transporters important for muscle metabolism, as well as the mitochondrial biogenesis inducer PGC-1*α*.^17^ Furthermore, MEF2 has been demonstrated to regulate the expression of several microRNA clusters, including microRNA-133a (miR-133a), that operate during muscle differentiation and regulate mitochondrial function.^18, 19^ In skeletal muscle, mice harboring deletions in the miR-133a alleles display a severe myopathy, accompanied by impaired mitochondrial respiration.^19^ Furthermore, miR-133a has been shown to regulate smooth muscle phenotype by altering proliferation.^20^

Diacylglycerol levels are chronically elevated in muscle tissues of obese and diabetic rodent models, and contribute to the lipotoxic and insulin-resistant state.^21, 22^ Therefore, one possible intracellular signaling pathway linking muscle metabolism with myogenesis is the diacylglycerol-protein kinase C (PKC)*δ* pathway. This pathway has been implicated in aberrant vascular smooth muscle growth, and can be viewed as an integrator of both metabolic and mitogenic cues.^23^ Interestingly, in human neonatal fibroblasts, PKC*δ* can inhibit SRF function by direct phosphorylation of threonine-160, which impairs SRF DNA binding leading to cell senescence.^24^ Furthermore, PKC*δ* signaling is reinforced by the proteolytic cleavage of a small constituently active PKC*δ* catalytic fragment from full-length PKC*δ*.^24^ However, whether this pathway regulates MEF2 or SRF in muscle tissues is untested.

In this report, we present the findings of an unbiased bioinformatics screen, utilizing position weight matrices. This computational approach predicted the co-occurrence of *cis* elements and a functional interaction between MEF2 and SRF. Experimentally, we demonstrate that MEF2C and SRF cooperatively activate the expression of miR-133a. Furthermore, we identify a conserved MADS-box phosphorylation motif, targeted by PKC*δ*, that serves to regulate endogenous miR-133a expression and mitochondrial function in all three muscle lineages. Finally, our data reveal that this signaling module is regulated by lipotoxicity to control mitochondrial function through the regulation of Nix, a known mitophagy and programmed cell death mediator.^25^

## Results

### Computational screen to predict transcription factor cooperation during muscle differentiation

To identify factors that collaborate with MEF2 proteins during muscle differentiation, we performed a bioinformatics screen that combined position weight matrices, conservation index, and optimized matrix threshold approaches. Since combinations of transcription factors regulate gene expression through their respective *cis* elements, this computational approach is founded on the hypothesis that one could predict functionally interacting factors based on the co-occurrence of their *cis* elements, within evolutionary conserved genomic regions. This analysis revealed that MEF2 is predicted to have target genes in common with seven other transcription factors (Supplementary Table 1). Among these was a predicted functional interaction between MEF2 and SRF. Since both MEF2 and SRF contain MADS-box domains, we investigated the hypothesis that MEF2 and SRF functionally cooperate during muscle differentiation and that this cooperation is regulated by a common intracellular signaling pathway.

### MEF2 and SRF cooperatively activate selective muscle-specific promoters

To experimentally validate the results of our bioinformatics screen, we initially studied the activation of the muscle creatine kinase (MCK) promoter as an index of muscle gene expression.^13^ We also evaluated representative cardiac (atrial natriuretic factor, ANF) and smooth muscle (telokin) promoters. As predicted by our bioinformatics screen, MEF2A and SRF cooperatively activated these promoters in Cos7 cells (Supplementary Figure 1). Next, we systematically engineered mutations in these promoters in order to understand how preventing MEF2 or SRF binding impacts promoter activity. For these experiments, the promoters were transfected into C2C12 cells, H9c2 cells, or a senescent-resistant human airway smooth muscle cell line (hASMC) to represent skeletal, cardiac, and smooth muscle myoblasts. Mutation of either the MEF2 or SRF *cis* element reduced the activity of the MCK, ANF, and telokin promoters (Supplementary Figure 1). Interestingly, mutation of the MEF2 *cis* element rendered the ANF and telokin reporter genes less responsive to mutation of the SRF site. Furthermore, mutation of all three *cis* elements simultaneously in the MCK promoter did not reduce promoter activity more than mutation of either MEF2 site alone. Collectively, these observations demonstrate a degree of functional dependency between MEF2 and SRF in the activation of these promoters in three different muscle cell lines.

### MEF2C and SRF regulate the endogenous expression of miR-133a

**Figure 1:**
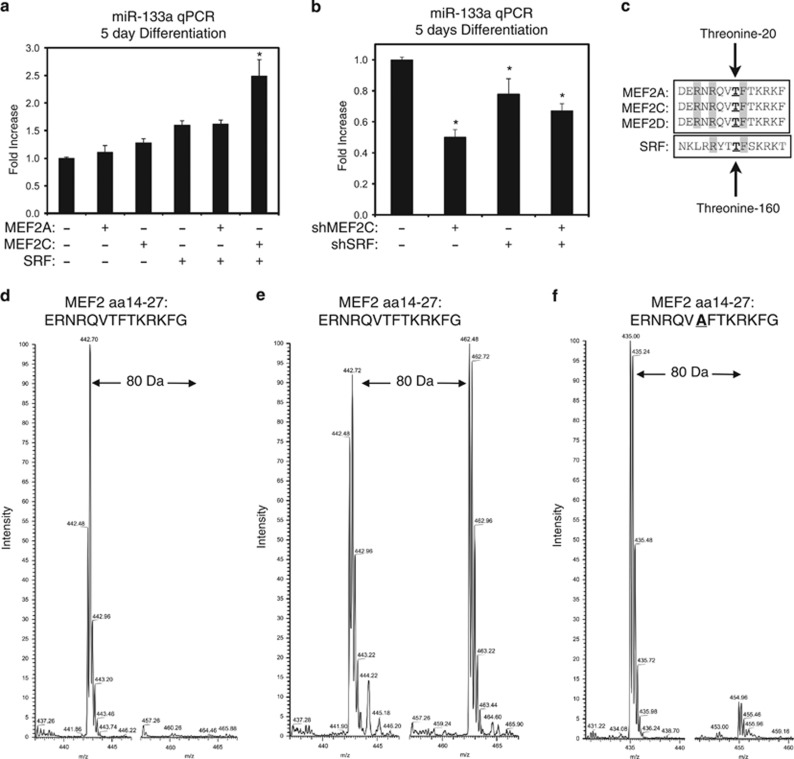
PKC*δ* inhibits the cooperation between MEF2 and SRF by direct phosphorylation. (**a**) C2C12 myoblasts were transfected with MEF2A, MEF2C, or SRF, as indicated. Following recovery, cells were differentiated in low serum media for 5 days and harvested for RNA. Quantitative PCR assays were performed using the ΔΔCT method, where RNU6 was used as an internal control. (**b**) C2C12 cells were transfected with shRNAs targeting MEF2C (shMEF2C) or SRF (shSRF), as indicated. Following 5 days of differentiation, cells were harvested and assayed as described above. (**c**) Schematic demonstrating the conservation surrounding the MEF2 threonine-20 and SRF threonine-160 phosphorylation motif. (**d**–**f**) SIM scans of the wild-type peptide (**d** and **e**) spanning the MADS-box motif of MEF2A. The unphosphorylated peptide (left) has 442 *m/z*, while the putative phosphorylation (right in **b**) showing an increased *m/z* of 20 that corresponds to PO_3_ (*M*=80.00 Da). (**f**) A SIM scan of a mutated peptide where threonine-20 is replaced with alanine is shown. On the right, phosphorylation of this mutate peptide is negligible at the predicted *m/z* that corresponds to the addition of a PO_3_ (*M*=80.00 Da). Both peptides are quadruply charged (*z*=4^+^). Data are represented as mean±S.E.M. *P<0.05 compared with control

Next, we focused our studies on the endogenous expression of a single MEF2 and SRF target gene that is expressed in all muscle lineages. For this we chose miR-133a, given that it has been recently identified as a regulator of muscle growth and metabolic function.^18, 19, 20^ We began with a gain-of-function approach, where C2C12 myoblasts were transfected with MEF2A, MEF2C, and SRF, alone and in combination. The combination of MEF2C and SRF induced endogenous miR-133a expression in differentiating C2C12 myotubes (Figure 1a), and we confirmed that ectopic expression of MEF2C and SRF was maintained at this timepoint (Supplementary Figure 2). Importantly, either factor alone had no effect, and MEF2A did not activate miR-133a expression. Interestingly, miR-133a has been shown to target both SRF and MEF2C, which suggests an element of feedback controlling the expression of this microRNA.^26, 27^ Next, C2C12 myoblasts were transfected with plasmids encoding short-hairpin RNAs (shRNAs) targeting MEF2C and SRF. Knockdown of MEF2C or SRF individually reduced the endogenous expression of miR-133a in differentiating myotubes (Figure 1b). Interestingly, simultaneous knockdown of both MEF2C and SRF did not additively reduce miR-133a expression (Figure 1b). Furthermore, knockdown of MEF2C and SRF was confirmed by qPCR at this timepoint (Supplementary Figure 2). These findings suggest a degree of functional dependency between MEF2C and SRF in the regulation of miR-133a expression that is consistent with our promoter analysis in Supplementary Figure 1.

### Peptide mapping of PKC*δ* phosphorylation of MEF2 and SRF by mass spectrometry

Previously, it was demonstrated that PKC*δ* phosphorylates SRF at threonine-160 to inhibit DNA binding.^24^ Since threonine-160 lies within the MADS-box of SRF, we aligned the amino-acid sequence of the MEF2 and SRF MADS-domains and found a high degree of conservation surrounding this phosphorylation site (Figure 1c), suggesting that this is a conserved MADS-box phosphorylation motif. To determine whether PKCδ directly phosphorylates this conserved MADS-box motif in MEF2 proteins, we performed *in vitro* kinase assays using engineered peptides representing amino acids 14–27 of MEF2. Following exposure to a kinase reaction with purified PKC*δ*, peptides were analyzed by mass spectrometry. As shown in Figure 1d, a single ion monitoring (SIM) scan of the the control peptide displayed a predominant peak at *m/z* of 442.74 (*z*=4^+^); however, following kinase incubation the peptide showed an increased *m/z* of 20, corresponding to the addition of a phosphate (PO_3_) to the peptide (Mass=80.00 Da; Figure 1e). Although an 80-Da mass shift is consistent with phosphorylation, it is neither unequivocal, nor does it permit localization of the phosphorylated amino acid. For location, we engineered an additional peptide where the residue representing threonine-20 was mutated to a neutral alanine. Mutation of threonine-20 substantially reduced the 80-Da mass shift (Figure 1f). These findings support the notion that threonine-20 is phosphorylated by PKC*δ*.

**Figure 2:**
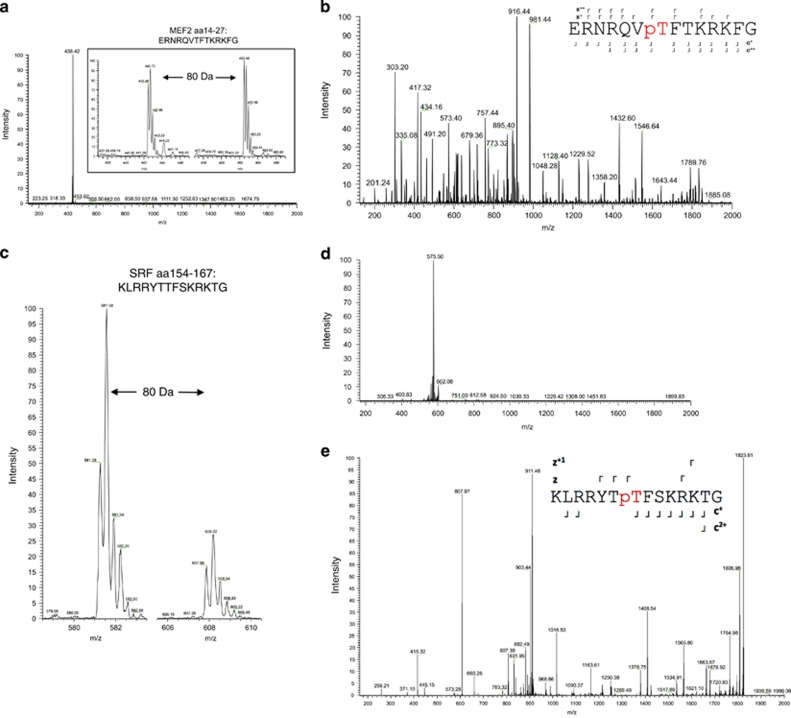
Identification of threonine-20 as a putative PKC*δ* phosphorylation residue. (**a**) Inset: SIM scan of native peptide (442.72, *z*=4^+^) and putative phospho-peptide (462.48, *z*=4^+^), as shown in Figure 1. The mass shift of +19.76 *m/z* is consistent with that of a quadruply charged peptide ion. Both threonine-20 and threonine-22 are possible sites of phosphorylation. Main figure: CID MS^2^ spectrum of the native peptide showing a prominent neutral loss ion at 438.42 *m/z* with *z*=4^+^. This represents a −24.56 Da shift consistent with a neutral loss of phosphate from threonine-20 or threonine-22 of the native peptide. (**b**) Fragmentation of the phospho-peptide (462.48, *z*=4^+^) by ETD produced a near-complete c and z ion series with some y ions also present. Analysis of this fragmentation spectra confirmed that threonine-20 is the preferred phosphorylation residue. Inset: Schematic illustrating the z and c ions detected by ETD. (**c**) SIM scan of a peptide spanning the MADS-box motif of SRF (amino acids 154–167). The unphosphorylated peptide (left) has 581.58 *m/z* (*z*=3^+^), while the putative phosphorylation (right in **a**, *m/z* of 608.22) showing an increased *m/z* of 26.64 that corresponds to PO_3_ (*M*=80.00 Da). (**d**) CID MS^2^ spectrum of the phospho-peptide in (**c**) with *m/z* of 608.22, showing a prominent neutral loss ion at 575.5 *m/z* with *z*=3^+^. (**e**) ETD MS^2^ spectrum of SRF confirming phosphorylation of threonine-160

Next, we analyzed the MS^2^ spectra produced by collision-induced dissociation (CID) of the precursor ion with *m/z*=462.98 (*z*=4^+^; shown in Figure 2a). CID typically fragments phospho-peptides (pS or pT) by breaking the labile O-phosphodiester bond. This results in the neutral loss of H_3_PO_4_, and a prominent neutral loss product-ion dominates the resulting MS^2^ spectrum. CID of the wild-type phospho-peptide yielded a product-ion with *m/z*=438.42 (delta=24.56), consistent with phosphorylation (98.0/4=24.5; Figure 4a). Furthermore, the precursor ion (*m*/*z*=462.98, *z*=4^+^) was selected for electron transfer dissociation (ETD). This technique breaks peptide bonds, but retains side-chain modifications, such as phosphorylation. MS^2^ spectra following ETD definitively identified threonine-20 of MEF2 as the phosphorylation residue (Figure 2b). Similar findings were observed for peptides spanning threonine-160 of SRF (Figures 2c–e).

### Site-directed mutagenesis of the PKC*δ* phospho-acceptor site on MEF2 and functional analysis

**Figure 3:**
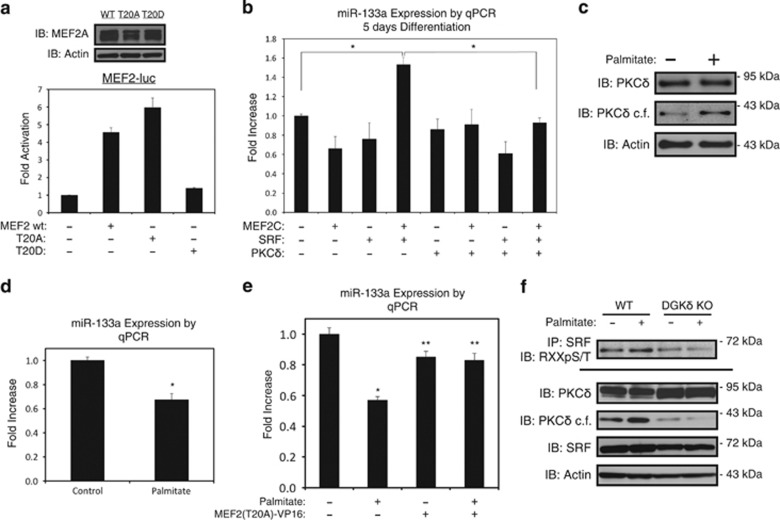
Mutational analysis of threonine-20. (**a**) HEK293 cells were transfected with MEF2A (MEF2 wt), or a plasmid containing MEF2A where threonine-20 is mutated to a neutral alanine (T20A) or phospho-mimetic aspartic acid (T20D), as indicated, and subjected to western blot (above). 10T1/2 cells were transfected, as above, along with MEF2-driven luciferase reporter gene (MEF2-luc). Extracts were subjected to luciferase assay, where *β*-galactosidase assay was used to correct for transfection efficiency (below). All assays were done in triplicate. (**b**) C2C12 myoblasts were transfected with MEF2C, SRF, or PKC*δ*, as indicated. Following recovery, cells were differentiated in low serum media for 5 days, harvested for RNA, subjected to qPCR assay. (**c** and **d**) Following 5 days of differentiation, C2C12 myotubes were treated with 200 *μ*M palmitate conjugated to 2% albumin in low glucose media overnight. Control cells were treated with 2% albumin alone. Myotubes were harvested for RNA or protein, and assayed by immunoblot (**c**) or qPCR (**d**). (**e**) C2C12 myoblasts were transfected with MEF2-VP16 fusion where threonine-20 is mutated to a neutral alanine [MEF2(T20A)-VP16], or control plasmid. Cell was differentiated for 5 days and treated with 200 *μ*M palmitate, as indicated. Myotubes were harvested for RNA and assayed by qPCR. (**f**) Wild-type (WT) or diacylglycerol kinase-*δ* knock-out (DGK*δ* KO) embryonic fibroblasts treated with palmitate, as described above. Extracts were immunoprecipitated (IP) with SRF antibody and probed using an antibody that recognizes phospho-serines/threonines with arginines at the –3 position (RXXpS/T) or immunoblotted (IB), as indicated. PKC*δ*=catalytic fragment of PKC*δ*. Data are represented as mean±S.E.M. *P<0.05 compared with control. **P<0.05 compared with palmitate treatment

To ascertain the cellular significance of PKC*δ*-dependent phosphorylation of threonine-20 of MEF2 and threonine-160 of SRF, we generated neutral alanine (MEF2A-T20A and SRF-T160A) and phospho-mimetic aspartic acid (MEF2A-T20D and SRF-T160D) mutations at these residues. As shown in Figure 3a, the MEF2A-T20A mutation displayed a modest increase in activity compared with wild-type MEF2A on a concatemerized MEF2-driven promoter (MEF2-luc), while the phospho-mimetic MEF2-T20D mutant provided no activation of this reporter construct. The expression of these mutants was confirmed by western blot to ensure that the introduced mutations did not alter MEF2 stability (Figure 3a). Similar findings were observed using a MCK-GFP reporter gene in differentiating C2C12 cells (Supplementary Figure 3c). Furthermore, we generated alanine and aspartic acid mutations of threonine-20 in a MEF2-VP16 fusion construct, where the MADS-box and adjacent MEF2 domain (amino acids 1–91 of MEF2A) are fused to the viral VP16 transcriptional activation domain.^23, 28^ These expression plasmids demonstrated similar activation pattern in a luciferase assay as the MEF2A mutations (Supplementary Figure 3). Finally, we determined that the phosho-mimetic mutations in MEF2A and SRF could disrupt the functional cooperation between these factors on the telokin promoter (Supplementary Figure 3D).

Next, we evaluated whether the endogenous expression of miR-133a by MEF2C and SRF was regulated by PKC*δ*. When the catalytic fragment of PKC*δ* was co-expressed with MEF2C and SRF, the induction of miR-133a was inhibited in differentiating C2C12s (Figure 3b). To determine the effect of an endogenous activator of PKC*δ* on miR-133a expression, we exposed cells to the saturated fatty acid palmitate.^21^ This treatment increased the active catalytic fragment of PKC*δ* (Figure 3c). Concurrently, we observed reduced expression of miR-133a (Figure 3d). Furthermore, when C2C12 cells were transfected with T20A-VP16, palmitate treatment was unable to inhibit miR-133a expression (Figure 3e).

To further define the role of diacylglycerols and PKC*δ* activation in the phosphorylation of the MADS-box motif, we utilized mouse embryonic fibroblasts genetically deficient in diacylglycerol kinase-*δ* (DGK*δ*).^29^ These cells display defective lipogenesis and reduced levels of diacylglycerols.^29^ Compared with wild-type cells, DGK*δ*-null fibroblasts have reduced PKC*δ* activity, determined by expression of the active catalytic fragment, in both vehicle- and palmitate-treated conditions (Figure 3f). Furthermore, we observed that phosphorylation of threonine-160 of SRF is reduced in the DGK*δ*-null fibroblasts, and that phosphorylation of this site is not enhanced by exposure to palmitate, as it is in wild-type fibroblasts (Figure 3f). These findings strongly implicate *de novo* diacylglycerol production and subsequent PKC*δ* activation in the phosphorylation of this MADS-box motif.

### Palmitate-induced mitochondrial depolarization involves PKC*δ* and inhibition of miR-133a expression

**Figure 4:**
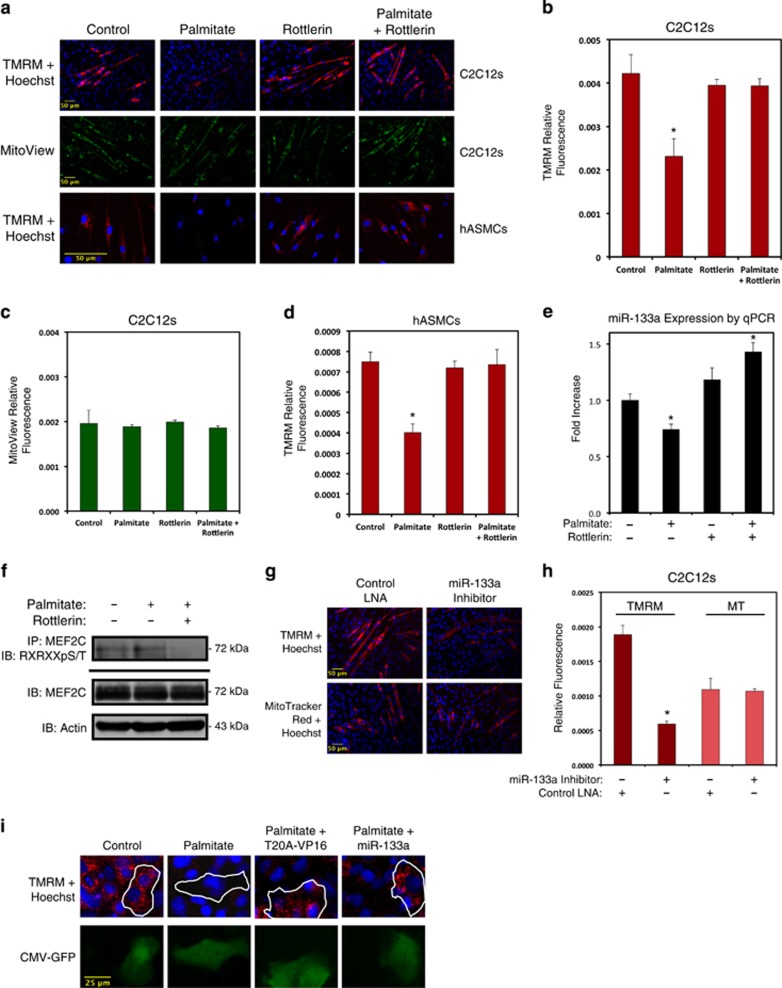
Palmitate-induced PKC*δ* activation regulates mitochondrial membrane potential through miR-133a. (**a**) C2C12 myotubes were differentiated for 5 days (above), or hASMCs were differentiated for 2 days (below), and treated with 200 *μ*M palmitate conjugated to 2% albumin in low glucose media with or without rottlerin (5 *μ*M) overnight. Control cells were treated with 2% albumin alone. Cells were stained with TMRM, Hoechst, and MitoView Green, as indicated, and imaged by standard fluorescence techniques (20 × for C2C12s; 40 × for hASMCs). (**b**–**d**) Fluorescent intensities from myotubes in (**a**) were quantified using ImageJ software (NIH). (**e**) hASMCs were treated as described in (**a**), harvested for RNA and subjected to qPCR analysis for miR-133a. (**f**) Differentiated C2C12 myotubes were treated as in (**a**). Protein extracts were immunoprecipitated (IP) with an MEF2C antibody, and probed using an antibody that recognizes phospho-serines/threonines with arginines at the –5 and –3 positions (RXRXXpS/T), and immunoblotted (IB), as indicated. (**g**) C2C12 cells were transfected with a miR-133a inhibitor oligonucleotide or a control oligonucleotide. Cells were differentiated for 5 days and imaged at 20 × with TMRM, Hoechst, or MitoTracker Red CMXRos, as indicated. (**h**) Quantification of myotube fluorescence in (**g**). (**i**) H9c2 myoblasts were transfected with MEF2-T20A-VP16 (T20A-VP16) or miR-133a. Following 2 days of differentiation, cells were treated with 200 *μ*M palmitate conjugated to 2% albumin or 2% albumin alone as a control. CMV-GFP was included to visualize transfected cells. Data are represented as mean±S.E.M. *P<0.05 compared with control

Given the recently described role of miR-133a in regulating muscle mitochondrial respiration,^19^ we hypothesized that palmitate-induced mitochondrial dysfunction involves PKC*δ*-dependent miR-133a inhibition. To test this hypothesis, we differentiated C2C12 cells and hASMCs, followed by overnight treatment with palmitate, and stained cells with fluorescent mitochondrial dyes. Palmitate treatment reduced TMRM staining in both cell lines (Figure 4a). Interestingly, palmitate treatment had little effect on MitoView Green staining in C2C12 myotubes, indicating a loss of mitochondrial membrane potential without a substantial loss of mitochondrial content (Figures 4a–c). However, treatment of C2C12 cells and hASMCs with the PKC*δ* inhibitor rottlerin abrogated the palmitate-induced loss of mitochondrial membrane potential (Figures 4a–c). Correspondingly, palmitate treatment reduced miR-133a expression, which was reversed by PKC*δ* inhibition by rottlerin (Figure 4d). Finally, we evaluated MEF2C phosphorylation at threonine-20 by immunoprecipitating endogenous MEF2C and western blotting the eluted proteins with a phospho-specific antibody that recognizes phospho-serines or phospho-threonines with arginine residues at the –3 and –5 positions (RXRXXpS/T).^30^ As shown in Figure 4f, palmitate exposure increased MEF2C phosphorylation, which was reversed by rottlerin treatment.

To assess the role of miR-133a in palmitate-induced mitochondrial depolarization mechanistically, we transfected C2C12 myoblasts with an inhibitory oligonucleotide targeting miR-133a. Control cells were transfected with a scrambled oligonucleotide. Following differentiation, cells were stained with either TMRM or MitoTracker Red CMXRos. As shown in Figure 4e, myotubes transfected with the miR-133a inhibitor displayed a reduced TMRM fluorescence, but an equivocal MitoTracker fluorescence, compared with control myotubes (Figures 4g and h). Next, we transfected myoblasts with a plasmid encoding miR-133a in order to reconstitute this microRNA's function in palmitate-treated cells. As shown in Figure 4i, cells expressing miR-133a were resistant to palmitate-induced disruption of mitochondrial membrane potential. We also transfected cells with T20A-VP16. Reconstitution of MEF2 function by this construct also enabled cells to be resistant to palmitate-induced mitochondrial depolarization (Figure 4i).

### miR-133a regulates mitochondrial function through the mitophagy and death gene Nix

**Figure 5:**
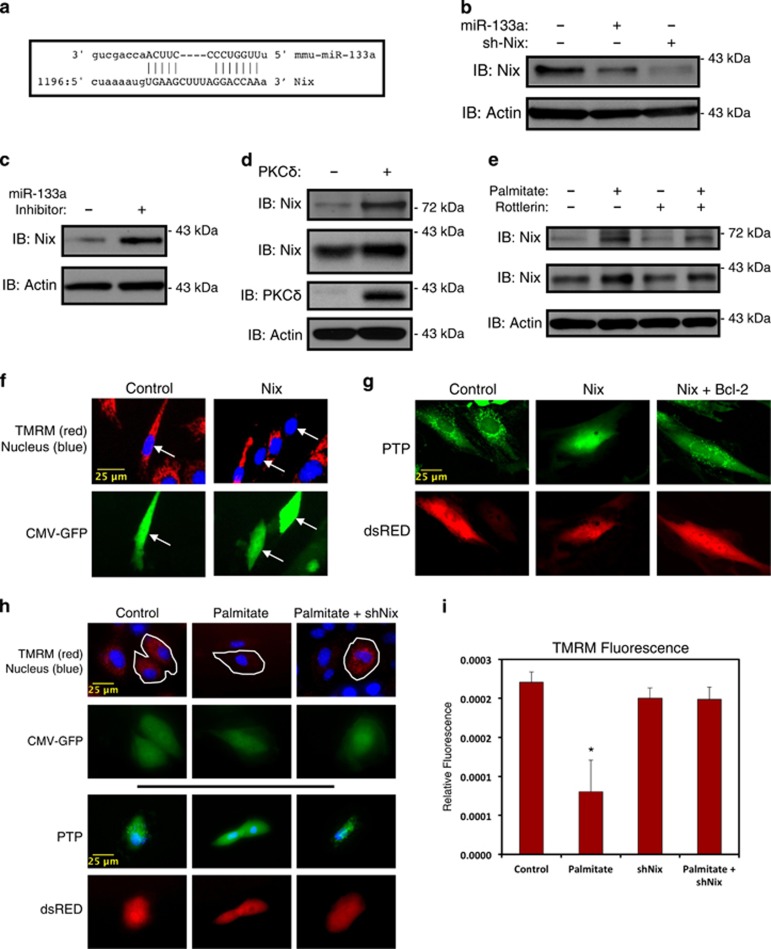
miR-133a regulates mitochondrial membrane potential through Nix. (**a**) Sequence alignment of mouse miR-133a and the 3' UTR of Nix. (**b**) H9c2 myoblasts were transfected with miR-133a or a shRNA targeting Nix (sh-Nix). Following protein extraction, samples were immunoblotted as indicated. (**c**) H9c2 cells were transfected with a miR-133a inhibitor (50 *μ*M) or a scrambled control oligonucleotide. Extracts were immunoblotted, as indicated. (**d**) C2C12 cells were transfected with the catalytic isoform of PKC*δ*. Extracts were immunoblotted, as indicated. (**e**) Five-day differentiated C2C12 cells were treated with 200 *μ*M palmitate conjugated to 2% albumin, or 5 *μ*M rottlerin overnight, as indicated. Protein extracts were immunoblotted, as indicated. (**f**) C2C12 myoblasts were transfected with Nix, or an empty vector control. CMV-GFP was included to identify transfected cells. Cells were stained with TMRM and Hoechst and imaged by standard fluorescence microscopy. Arrows indicate GFP-positive cells. (**g**) H9c2 cells were transfected with Nix and Bcl-2, as indicated. CMV-dsRed was used to identify transfected cells. Cells were stained with calcein-AM with cobalt chloride (5 *μ*M) to assess PTP opening. (**h**) H9c2 cells were transfected with shNix or a scrambled control shRNA. Following recovery, cells were treated with 200 *μ*M palmitate conjugated to 2% albumin overnight, and stained with TMRM and Hoechst to evaluate mitochondrial membrane potential (above) or with calcein-AM with cobalt chloride (5 *μ*M) to assess PTP opening (below). (**i**) Quantification of myotube fluorescence in (**h**). Data are represented as mean±S.E.M. *P<0.05 compared with control

To identify a mechanism by which miR-133a regulates mitochondrial function, we performed an *in silico* screen in an attempt to identify novel mRNA targets of miR-133a. This screen identified a conserved miR-133a target sequence in the 3'-untranslated region of the human and rodent Nix mRNA (Figure 5a). Thus, we expressed miR-133a in cultured myoblasts, and observed reduced protein expression of Nix, where a shRNA targeting Nix (shNix) was used as a positive control (Figure 5b). In addition, when miR-133a was expressed in myoblasts, we did not detect a change in Nix mRNA, suggesting that miR-133a inhibits Nix expression by translational block rather than mRNA degradation. To perform the reciprocal experimentation, we utilized a miR-133a inhibiting oligonucleotide, and observed increased expression of Nix protein (Figure 5c). Previous studies have shown that Nix can form a SDS-resistant dimer with a predicted molecular weight of 80 kDa.^31^ Ectopic expression of the catalytic fragment of PKC*δ* increased the protein expression of both monomeric and dimeric Nix (Figure 5d). Consistent with these findings, overnight palmitate treatment increased the protein expression of both monomeric and dimeric Nix, which was reversed by the PKC*δ* inhibitor rottlerin (Figure 5e). To investigate the role of Nix in mitochondrial function, we transfected C2C12 cells with Nix, and stained cells with TMRM. We observed reduced mitochondrial membrane potential in C2C12 myoblasts that expressed Nix compared with control (Figure 5f). In addition, we observed that expression of Nix opened the mitochondrial permeability transition pore (PTP) when transfected into the cardiac H9c2 myoblasts, as evident by the loss of green mitochondrial puncta, where this effect was reversed by co-expression of the pro-survival gene Bcl-2 (Figure 5g). Finally, to establish the role of Nix in palmitate-induced mitochondrial dysfunction, we transfected H9c2 myoblasts with shNix and evaluated mitochondrial PTP opening and membrane potential following palmitate exposure. As shown in Figure 5h, exposure to palmitate opened the mitochondrial PTP and reduced mitochondrial membrane potential; however, the shNix restored both mitochondrial puncta, and reversed the effect of palmitate on mitochondrial membrane potential (Figure 5i).

**Figure 6:**
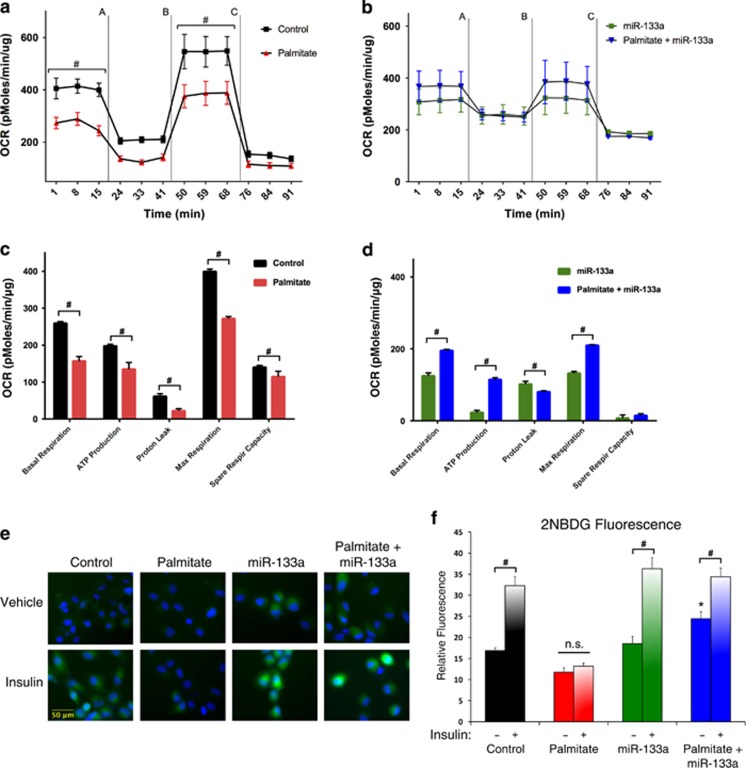
Evaluation of mitochondrial respiration and glucose uptake. (**a**) Differentiated H9c2 cells were treated overnight with 200 *μ*M palmitate conjugated to 2% albumin in low glucose media. Control cells were treated with 2% albumin alone. Oxygen consumption rate (OCR) was evaluated on a Seahorse XF-24. To evaluate mitochondrial function, cells were injected with oligomycin (1* μ*M) (**a**), FCCP (1 *μ*M) (**b**), and antimycin A (1 *μ*M) and rotenone (1 *μ*M) (**c**). (**b**) H9c2 cells were transfected with a miR-133a mimic (50 *μ*M) or a scrambled control oligonucleotide. Following recovery, OCR evaluated as in (**a**). (**c** and **d**) Calculated respiration rates from (**a**) and (**b**), respectively. (**e** and **f**) H9c2 cells were transfected as in (**b**) and treated as in (**a**). Insulin stimulated uptake (10 nM) was determined by 2NBDG fluorescence and quantified in (**f**). Data analyzed by two-way ANOVA, and represented as mean±S.E.M. ^#^*P*<0.05 between groups, *P<0.05 compared with control

To determine the role of miR-133a in mitochondrial physiology, we evaluated oxygen consumption rate in cultured H9c2 cells. As shown in Figure 6a, overnight exposure to palmitate reduced oxygen consumption, as well as the calculated basal and maximum respiration rates when cells were stressed with oligomycin (a), FCCP (b), and antimycin A and rotenone (c) (Figure 6c).^32^ However, when cells were transfected with a miR-133a mimicking oligonucleotide, the palmitate-induced drop in oxygen consumption was prevented (Figure 6b), where control cells were transfected with a scrambled oligonucleotide. Furthermore, the calculated basal and maximum respiration rates were increased when miR-133a mimic-treated cells were exposed to palmitate (Figure 6d), suggesting that improved mitochondrial function enabled cells to metabolize the available palmitate. Finally, we determined whether restoration in mitochondrial function translated into improved insulin-stimulated glucose uptake. As shown in Figures 6e and f, overnight exposure to palmitate prevented insulin-stimulated glucose uptake in differentiated H9c2 cells, determined by the fluorescence glucose analog 2NBDG, which was restored when cells were treated with a miR-133a mimic. Interestingly, basal glucose uptake was also increased in cells treated with the miR-133a mimic and exposed to palmitate.

### Evaluation of miR-133a and Nix expression *in vivo*

**Figure 7:**
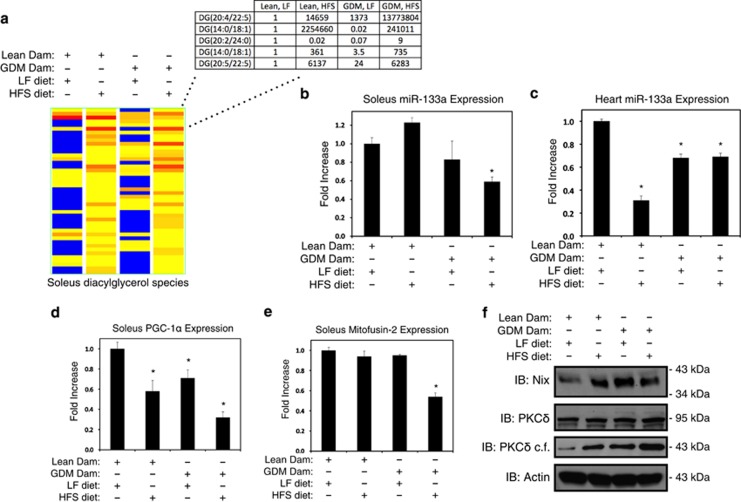
miR-133a expression *in vivo*. (**a**) Metabolomics analysis of 44 species of diacylglycerols from soleus muscle excised from LF, or HFS, and a normal pregnancy (Lean Dam), or gestational diabetes (GDM Dam) during development, as indicated. Five highly abundant diacylglycerols are highlighted in the chart. Blue=low abundance, yellow=medium low abundance, orange=medium high abundance, red=high abundance. (**b**–**e**) Rat soleus muscle or heart tissue was excised and total RNA was extracted, from rodents treated as described in (**a**). qPCR analysis was performed using the ΔΔCT method, where RNU6 was used as an internal control for miR-133a, and *β*-actin was used as a control for PCG-1*α* and mitofusin-2. (**f**) Protein extracts from rat soleus muscle were subjected to immunoblot analysis, as indicated. PKC*δ* catalytic fragment (PKC*δ* c.f.). Data are represented as mean±S.E.M. *P<0.05 compared with control

To determine the *in vivo* relevance of this genetic pathway in muscle tissues, we utilized a rodent model of gestational diabetes, as fetal exposure to diabetes during pregnancy increases the risk for early-onset insulin resistance in the offspring and may program metabolism.^4, 5^ Rats exposed to diabetes during gestation become insulin resistant by 15 weeks of age, a phenotype that is exacerbated by the postnatal consumption of a high-fat and sucrose (HFS) diet.^5^ To further define the metabolic alterations induced by exposure to gestational diabetes in muscle tissue, we performed metabolomics analysis by mass spectrometry using extracts enriched for lipid-soluble metabolites. This screen identified 44 species of diacylglycerols varying in the composition of their fatty acid chains (Supplementary Table 2). In the offspring of normoglycemic lean dams, numerous diacylglycerol species were increased by the HFS diet (Figure 7a). However, exposure to maternal diabetes increased diacylglycerols in the soleus muscle of both low fat (LF) and HFS-fed offspring, suggesting the presence of a programming effect on soleus muscle (Figure 7a). Thus, we hypothesized that the accumulation of diacylglycerol species would provide an activating stimulus for PKC*δ*. In support of this, we observed that miR-133a expression was reduced by 40% in the soleus and heart of animals exposed to both gestational diabetes and a HFS diet (Figures 7b and c). In addition, we evaluated the expression of mitochondrial marker genes in the soleus muscle of our rodent model. PGC-1*α* expression was reduced by both the postnatal consumption of the HFS diet and fetal exposure to gestational diabetes, and the consumption of HFS diets by the offspring of diabetic dams caused an additive suppression of PGC-1*α* expression (Figure 7d). Moreover, mitofusin-2 expression was also reduced in the gestational diabetes offspring fed HFS diets (Figure 7e). In addition, we observed an increased expression of Nix in the soleus of rats exposed to the HFS diet or gestational diabetes (Figure 7f), concurrent with increased expression of the catalytic fragment of PKC*δ*. These findings suggest that developmental programming of diacylglycerol metabolism and miR-133a expression influences mitochondrial markers, and Nix expression, in the offspring.

## Discussion

PKC signaling has been implicated in lipotoxicity and diabetic complications in multiple cell types,^22^ although the precise molecular mechanisms have not yet been defined in muscle tissues. Previously, our research group demonstrated that novel PKC isoforms could activate the C-terminus of MEF2 proteins utilizing Gal4-DNA binding domain fusion proteins.^33^ In addition, novel PKC isoforms activate a PKD-dependent signaling cascade resulting in liberation of MEF2 from HDAC5 repression during cardiac growth.^34^ However, during the course of our investigations, we observed that expression of PKC*δ* could inhibit MEF2 activity in some cellular contexts. Utilizing three independent muscle cell lines, an *in vivo* model, and mouse embryonic fibroblasts genetically deficient in DGK*δ*, we characterized a novel pathway initiated by PKC*δ* signaling during lipotoxicity that converges on MEF2 and SRF transcription factors to regulate muscle gene expression and mitochondrial membrane potential. Detailed mass spectrometry analysis revealed that PKC*δ* phosphorylates MEF2 proteins at threonine-20 and SRF at threonine-160, a conserved MADS-box residue. Downstream, collaborative regulation of miR-133a expression by MEF2C and SRF is attenuated by PKC*δ* phosphorylation of these transcription factors. Importantly, expression of either miR-133a or an active MEF2 fusion protein, which cannot be phosphorylated by PKC*δ*, reverses palmitate-induced mitochondrial dysfunction.

Recently, phosphorylation of *Drosophila* MEF2 at threonine-20 was shown to direct MEF2 activity between immune activation and metabolic function.^30^ In flies, MEF2 is normally phosphorylated at threonine-20 to promote anabolic gene expression. However, during infection MEF2 is dephosphorylated and targeted to genes involved with immune function at the expense of anabolic nutrient storage. At first, these results may seem contrary to the findings of the present study. However, regulation of MEF2 and SRF by phosphorylation at the conserved MADS-box motif, and its downstream effects on miR-133a and Nix, may have evolved to limit mitochondrial metabolism and divert nutrients to anabolic storage or cell growth during differentiation. Thus, the novel genetic pathway identified in the present study may represent an anabolic survival pathway, maintained through evolution in part due to the conservation of the MADS domain.

One of the most intriguing findings of the present study is the identification of Nix as a miR-133a target. Previous studies have implicated both Nix and miR-133a in the regulation of mitochondrial function and programmed cell death in multiple cell types,^19, 35, 36, 37^ and our data strongly suggest that miR-133a is dependent on Nix for mitochondrial membrane potential regulation in muscle tissues. Furthermore, both Nix and miR-133a are involved in pathological cardiac remodeling.^38, 39, 40^ Thus, our findings may represent an important mechanism involved in diabetes-induced heart disease and have an important role in the progression to heart failure following cardiac injury.

In summary, these studies document a novel signaling cascade triggered by lipotoxicity, and converging on MEF2 and SRF transcription factors to regulate the expression of miR-133a during muscle development and post-natal remodeling. Repression of miR-133a expression ultimately regulates mitochondrial function, through the Bcl-2 family member Nix, which may have implications to pathological states such as insulin resistance and cardiovascular disease.

## Materials and Methods

### Bioinformatics screen to predict functional transcription factor interactions

Muscle gene expression data were compiled from the profile published by Zhang *et al.*^41^ Genomic sequence representing the proximal promoter region (−1000 to +200 bp) of 46 muscle genes was extracted from the Database of Transcriptional Factor Start Sites (DBTSS) and used for this analysis. Position weight matrices of all known mouse transcription factors were collected from the TRANSFAC and Wasserman-Fickett databases. We determined the conserved index (Ci) by calculating the degree of conservation of individual nucleotides in the matrix as a numerical value. In this model, Ci varies between 0 and 100, where 100 represents a position with total conservation of one nucleotide, and 0 represents equal distribution of all four possible nucleotides. Using this approach, we defined a core binding region as a region with four consecutive nucleotide positions with the highest Ci values, and used this core binding region to reduce the number of matches in the position weight matrices. We calculated the optimized matrix threshold and screened the promoter regions of the target sequences with the position weight matrices and optimized matrix threshold to detect interacting partners of MEF2 proteins based on the co-occurrence of *cis*-regulatory elements.

### Plasmids

The MEF2 plasmids were described previously.^28, 42, 43^ The plasmid expressing the PKC*δ* catalytic fragment was kindly provided by K Wheaton. The miR-133a expression plasmid was purchased from Addgene (Principal Investigator David Bartel, plasmid 26326).^44^ The shRNA targeting SRF was based on the targeting sequence previously described by Medjkane *et al.* (5′-CTGCAGCCCATGATCACCA-3′).^45^ Sense and antisense oligonucleotides containing the target sequence were purchased from Sigma Aldrich (Oakville, ON, Canada), annealed, and ligated into pSilencer 3.0 H1 (Ambion, ThermoFisher, Burlington, ON, Canada). The Nix (Bnip3L), shNix, and Bcl-2 plasmids were purchased from Addgene (Principal Investigator Wafik El-Deiry, plasmids 17467 and 17469, and Principal Investigator Clark Distelhorst, plasmid 18003).^46, 47^

### Cell culture and transfections

All cell lines were maintained in Dulbecco's modified Eagle's medium (DMEM; Hyclone, Fisher Scientific, Ottawa, ON, Canada), containing penicillin, streptomycin, and 10% fetal bovine serum (Hyclone) at 37 °C and 5% CO_2_. Generation of the hTERT senescent-resistant hASMC was described previously,^48^ as were the DGK*δ*-null fibroblasts.^29^ C2C12 and hASMCs were transfected using JetPrime Polyplus reagent, and the H9c2 cell line was transfected using Qiagen's Polyfect reagent, as per the manufacturer's instructions. C2C12 was differentiated by re-feeding cells in 2% fetal bovine serum (Hyclone) for to 2–5 days, as indicated in the figure legends, while H9c2 cells were differentiated in 1% fetal bovine serum (Hyclone) for 24–48 h. Palmitate conjugation and treatments were performed as described by Chavez *et al.*^49^

### *In vitro* kinase assay

Synthetic peptides (Fisher Scientific) were resuspended in molecular biology grade water at a concentration of 1 mg/ml. Peptide sequences used were MEF2 wild-type amino acids 14–27: ERNRQVTFTKRKFG, MEF2 threonine-20 mutation: ERNRQV***A***FTKRKFG, SRF amino acids 154–167: KLRRYTTFSKRKTG. These peptides were used as the substrate in a PKC*δ* kinase assay kit (SignalChem, Richmond, BC, Canada) according to the manufacturer's instructions, with the exception that [^32^P]-ATP was replaced with fresh molecular biology grade ATP. The manufacturer's CREBtide synthetic peptide substrate (KRREILSRRPSYR) was used as a positive control in each assay. Following incubation at 30 °C for 15 min, reactions were frozen at −80 °C before mass spectrometry analysis.

### Phospho-peptide mapping

Before mass spectrometry analysis, kinase assays were prepared using C_18_ ZipTips (EMD Millipore, Etobicoke, ON, Canada), according to the manufacturer's protocol, to desalt and concentrate peptides. Samples in 50% acetonitrile and 0.1% formic acid were introduced into a linear ion-trap mass spectrometer (LTQ XL: ThermoFisher, San Jose, CA, USA) via static nanoflow, using a glass capillary emitter (PicoTip: New Objective, Woburn, MA, USA). All spectra were acquired using the ZoomScan setting. For MS^2^, CID or ETD was used. For the former, collision energy was set to 25% for the latter a reaction time of 100 ms with fluoranthane was set. Spectra were sequenced *de novo* manually.

### Immunoprecipitation and immunoblotting

Protein extractions were achieved using a RIPA lysis buffer containing protease inhibitors and phosphatase inhibitors (Santa Cruz, Dallas, TX, USA). Protein concentrations were determined using a Bio-Rad Canada (Mississauga, ON, Canada) Protein assay kit. Extracts were resolved using SDS-PAGE and transferred onto a PVDF membrane. Immunoblotting was carried out using appropriate primary antibody in 5% powdered milk or BSA in TBST. Appropriate horseradish peroxidase-conjugated secondary antibody (Jackson ImmunoResearch Laboratories, West Grove, PA, USA; 1 : 4000) was used in combination with chemiluminescence to visualize bands. Immunoprecipitations utilized the Immunocruz kit (Santa Cruz), described previously,^43^ and complexes were probed by immunoblot using the RXRXXpS/T or RXXpS/T phospho-antibodies from Cell Signaling Technology (Danvers, MA, USA).

### Fluorescent staining

MitoTracker Red CMXRos was purchased from Cell Signaling Technology and applied to cells for 30 min. Following incubation, cells were re-fed standard DMEM. TMRM, Calcein-AM, MitoView Green, and Hoechst 33342 were purchased from Biotium (Hayward, CA, USA). PTP imaging was performed by quenching the cytosolic Calcein-AM signal with 5 *μ*M cobalt chloride during the incubation period. All imaging was done on an Olympus IX70 inverted microscope (Toronto, ON, Canada) with QImaging Retiga SRV Fast 1394 camera (Surrey, BC, Canada) using NIS Elements AR 3.0 software (Nikon Instruments Inc., Melville, NY, USA). Quantification, scale bars, and processing were done on ImageJ software (NIH, Bethesda, MD, USA).

### Mitochondrial respiration and glucose uptake

Mitochondrial respiration was determined on a Seahorse XF-24 Extracellular Flux Analyzer (Seahorse Bioscience, North Billerica, MA, USA), as described previously.^32^ Calculated respiration rates were determined as per manufacturer's instructions (Mito Stress Kit; Seahorse Bioscience). Insulin-stimulated glucose uptake was evaluated in differentiated H9c2 cells incubated in the presence or absence of 10 nM insulin in phosphate-buffered saline (PBS) for 15 min, followed by 15 min with the inclusion of the fluorescent D-glucose analog 2NBDG (200 *μ*M; Molecular Probes, ThermoFisher). Cells were imaged by standard techniques and quantified using ImageJ.

### Diet-induced gestational diabetes model

All procedures in this study were approved by the Animal Welfare Committee of the University of Manitoba, which adheres to the principles for biomedical research involving animals developed by the Council for International Organizations of Medical Sciences. Female Sprague-Dawley rats were obtained at 4 weeks of age (University of Manitoba Colony) and randomly allocated to a LF diet (10% fat, Research Diets D12450B) or a HFS diet (45% fat, Research Diets D12451) for 6 weeks to induce pre-gestational glucose intolerance.^5, 50^ The female rats continued on their respective diets throughout pregnancy and weaning. The high-fat/sucrose-fed female rats developed hyperglycemia characteristic of gestational diabetes (GDM) while pregnant. Pups were weaned at 3 weeks of age and randomly assigned to either LF or HFS diets for 12 weeks, creating four experimental groups: the offspring of lean mothers fed LF or HFS diets and the offspring of GDM mothers fed LF or HFS diets. For tissue analysis, rats were euthanized by overdose of sodium pentobarbital, and the heart and soleus muscles were dissected, rinsed in PBS, and immediately clamp frozen in liquid nitrogen.

### Quantitative PCR

Total RNA was extracted from cultured cells and pulverized frozen tissue by the TRIzol method. For microRNA analysis, all primers were purchased from Quanta BioSciences (Gaithersburg, MD, USA). cDNA was generated using QScript MicroRNA cDNA Synthesis kit (Quanta BioSciences) and q-RT-PCR performed using PerfeCTa SYBR green super mix on a Applied Biosystems 7500 Real-Time PCR Instrument (ThermoFisher), and normalized to RNU6 expression. For mRNA analysis, following column purification using Qiagen RNeasy kit and DNase treatment (Qiagen, Toronto, ON, Canada), cDNA was generated with QScript cDNA super mix (Quanta BioSciences) and analyzed as described above, and normalized to *β*-actin expression. Primers used were PGC-1*α* total: Forward 5′-CAGCTTTCTGGGTGGATTGA-3′ and Reverse 5′-GCTCATTGTTGTACTGGTTGGA-3′, Mitofusin-2: Forward 5′-CTCTCAAGCACTTTGTCACTGC-3′ and Reverse 5′-TGTATTCCTGTGGGTGTCTTCA-3′, *β*-actin: Forward 5′-TTGCTGACAGGATGCAGAAG-3′ and Reverse 5′-TAGAGCCACCAATCCACACA-3′.

### Mass spectrometry metabolomics analysis

Analysis of total soleus lipids was performed using a lipid soluble extraction, and analysis was preformed as described previously.^5^ Briefly, lipid extracts were reconstituted with 100 μl of 80% acetonitrile prepared in deionized water. Metabolomics analysis was performed on a 1290 Infinity Agilent high-performance liquid chromatography (HPLC) system coupled to a 6538 UHD Agilent Accurate Q-TOF LC/MS equipped with a dual electrospray ionization source. A 3 × 50 mm, 2.7 μ Agilent Poroshell column was used to separate metabolites while the column temperature was maintained at 60 °C. The mass detection was operated using dual electrospray with reference ions of *m/z* 121.050873 and 922.009798 for positive mode; and *m/z* 119.03632 and 980.016375 for negative mode. The workflow utilized for data processing comprised several algorithms used by Agilent Mass Hunter Qualitative (MHQ, B.05, Agilent Technologies, Mississauga, ON, Canada) and by Mass Profiler Professional (MPP, 12.6, Agilent Technologies).

## Abbreviations

MEF2: myocyte enhancer factor-2
SRF: serum response factor
miR-133a: microRNA-133a
MADS domain-box: *M*cm1 *A*gamous *D*eficiens *S*RF
PKC*δ*: protein kinase C *δ*
HFS: high-fat and sucrose
Nix/Bnip3L: BCL2/adenovirus E1B 19 kDa protein-interacting protein 3-like
PTP: permeability transition pore
DGK*δ*: diacylglycerol kinase-*δ*
