# Supplementary material for: A conserved MADS-box phosphorylation motif regulates differentiation and mitochondrial function in skeletal, cardiac, and smooth muscle cells
Source: Cell Death Dis. 2015 Oct 29;6(10):e1944–. doi: 10.1038/cddis.2015.306 (PMC5399178; doi:10.1038/cddis.2015.306)
Supplement: Supplementary Figure 1 [file cddis2015306x1.pdf]

MATERIALS AND METHODS

Plasmids. The MEF2 plasmids were described previously[1-3]. The muscle creatine kinase promoter constructs were provided by A. Ferrer-Matinez. Mutations of the MEF2 and SRF *cis* elements were generated by site-directed mutagenesis to achieve the following sequence: MEF2-near wild-type 5'-CTAAAAATAA-3' mutant 5'-CT**GG**A**GG**T**CC**-3', MEF2-far wild-type 5'-GTTATAATTAA-3' mutant 5'-**C**T**G**ATA**G**T**AG**A-3', SRF wild-type 5'-CCATGTAAGG-3' mutant 5'-**AG**AT**C**TAA**CC**-3'. The ANF-700 bp luciferase promoter was a kind gift from M. Nemer. Mutations in the MEF2 and SRF *cis* elements are: MEF2 wild-type 5'-CTAAAAAAATA-3' mutant 5'-CTAA**GGG**AATA-3' and SRF wild-type 5'-CCTTATTTGG-3' mutant 5'-CCTTA**GGG**GG-3'. The 370 bp telokin promoter was gifted by P. Herring, and the following mutations were generated by using a site-directed approach: MEF2 wild-type 5'-CTTTATATAA-3' mutant 5'-CTTT**GG**ATAA-3' and SRF wild-type 5'-CCTTTTATGG-3' mutant 5'-CCTTTT**C**T**AC**-3'. The CMV-SRF and *c-fos* luciferase plasmids were provided by P. Shaw. The MASTR plasmid was generously provided by E. Olson.

Transcriptional response assays. For standard luciferase assays, Cos7, HEK-293, and C310T1/2 cells were transfected by calcium phosphate method.. pCMV-β-galactosidase was used as an internal transfection efficiency control. Following transfection, cells were washed twice in PBS or DMEM, and re-fed in standard media. Cells were lysed after a 48-hour recovery period and assayed according to manufacturer's instructions (Promega Luciferase assay kit) on a Berthold Luminometer, where β-galactosidase assay was used as a transfection efficiency control.

FIGURE LEGENDS

Supplemental Figure 1. MEF2A and SRF cooperatively active select muscle-specific promoters.
A-D. Cos7 cells were transfected with MEF2A and SRF, as indicated, along with luciferase-driven promoters for muscle creatine kinase (MCK-luc), atrial natriuretic peptide (ANP-luc), telokin (telokin-luc), or *c-jun*. Extracts were subject to luciferase assay, where β-galactosidase assay was used to correct for transfection efficiency. E-G. C2C12 (E), H9c2 (F) and hASMC (G) myoblasts were transfected with the wild-type (WT) promoters, or constructs where the SRF binding site is mutated (ΔCArG) or the MEF2 sites are mutated (ΔMEF2). Cells were allowed to differentiate for 2-days in low serum media prior to harvesting for luciferase and β-galactosidase assay. All assays were done in triplicate.

Supplemental Figure 2. Over-expression and knockdown control experiments.
A-B. C2C12 cells were transfected with MEF2C and SRF (Shown in Figure 1A). RNA extracts were analysed for MEF2C and SRF expression by qPCR to ensure over-expression was maintained following differentiation. C-D. C2C12 cells were transfected with shMEF2C and shSRF (Shown in Figure 1B). qPCR was performed to ensure knockdown was maintained throughout differentiation.

Supplemental Figure 3. Mutational analysis of Threonine-20.
A. 10T1/2 cells were transfected with MEF2A, SRF, or PKCδ, as indicated. Extracts were subject to luciferase assay, where β-galactosidase assay was used to correct for transfection efficiency. B. 293 cells were transfected with MEF2A-VP16 fusion protein, or a construct where threonine-20 is mutated to a neutral alanine (T20A-VP16) or phospho-mimetic aspartic acid (T20D-VP16), as indicated, along with MCK promoter (MCK-luc). Extracts were subject to luciferase assay as in (A). B. 293 cells were transfected with MASTR, MEF2A, and MEF2A-T20A (T20A), as indicated. Extracts were assayed as described above. C. C2C12 myoblasts were with MEF2A, MEF2A-T20A and MEF2A-T20D, as indicated, along with a MCK-GFP reporter-gene and CMV-dsRed to identify transfected cells. Following recover cells were differentiated in low-serum media for 96-hours and imaged by standard fluorescent techniques. D. 293 cells were transfected with MEF2A, SRF, or plasmids containing phospho-mimetic aspartic acid mutations in MEF2A and SRF (T20D and T160D), as indicated, along with the telokin promoter (Telokin-luc). Extracts were assayed as described above.

REFERENCES

1. Mcdermott JC, Cardoso MC, Yu YT, Andres V, Leifer D, Krainc D *et al.* hMEF2C gene encodes skeletal muscle- and brain-specific transcription factors. *Mol Cell Biol* 1993; **13**: 2564–2577.

2. Ornatsky OI, Andreucci JJ, Mcdermott JC. A dominant-negative form of transcription factor MEF2 inhibits myogenesis. *J Biol Chem* 1997; **272**: 33271–33278.

3. Perry RLS, Yang C, Soora N, Salma J, Marback M, Naghibi L *et al.* Direct interaction between myocyte enhancer factor 2 (MEF2) and protein phosphatase 1alpha represses MEF2-dependent gene expression. *Mol Cell Biol* 2009; **29**: 3355–3366.
