# Supplementary figures and images for: A conserved MADS-box phosphorylation motif regulates differentiation and mitochondrial function in skeletal, cardiac, and smooth muscle cells
Source: Cell Death Dis. 2015 Oct 29;6(10):e1944–. doi: 10.1038/cddis.2015.306 (PMC5399178; doi:10.1038/cddis.2015.306)

Supplemental Figure 1.

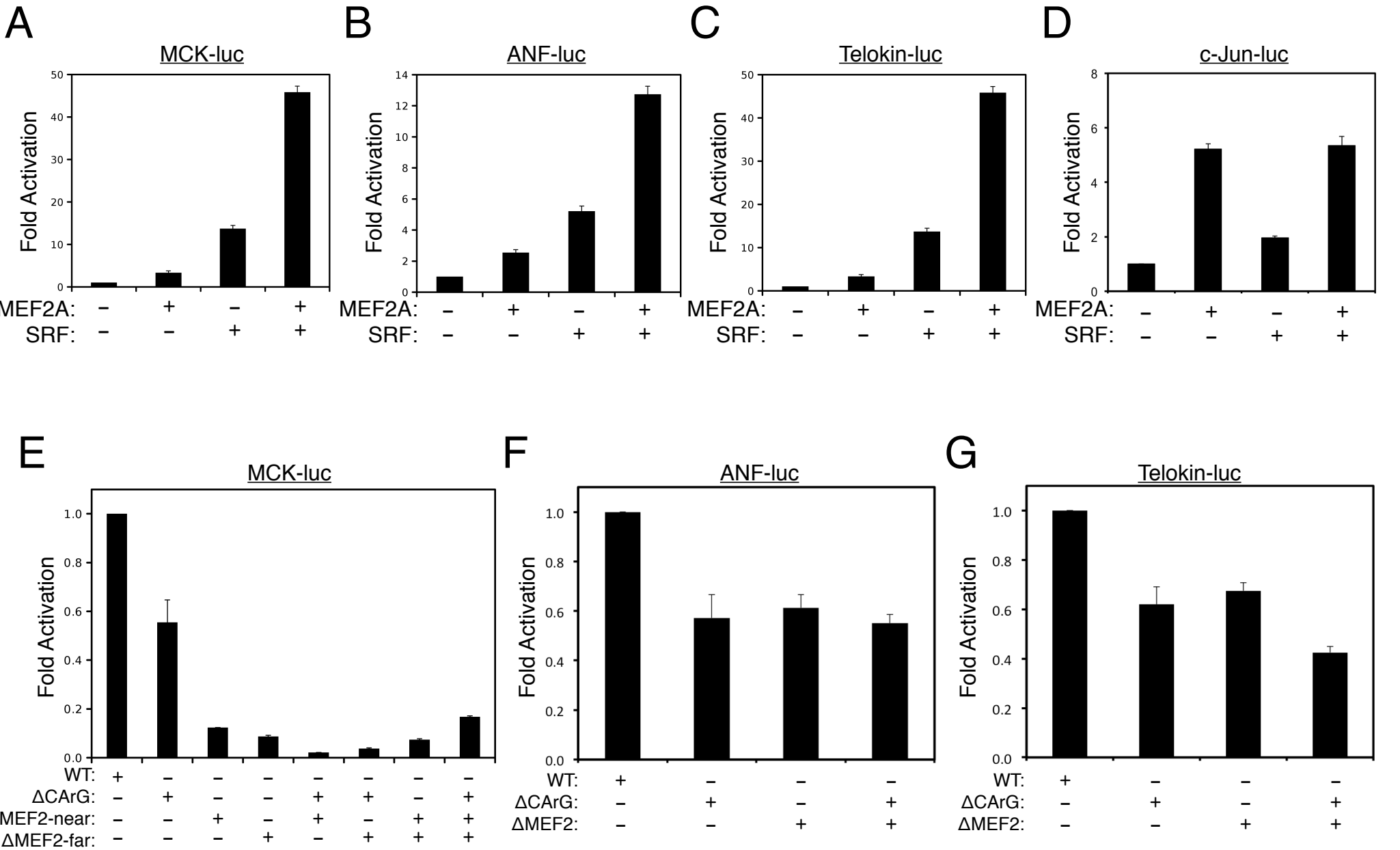

Supplement: Supplementary Figure 2 [file cddis2015306x2.pdf]

Supplemental Figure 2.

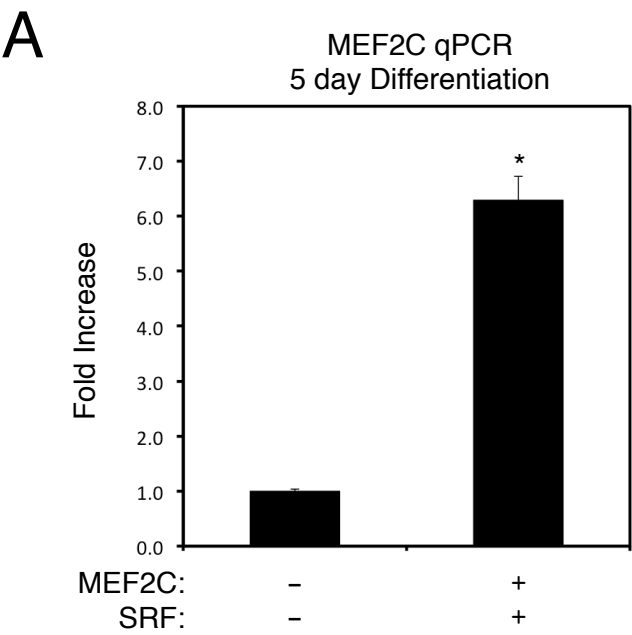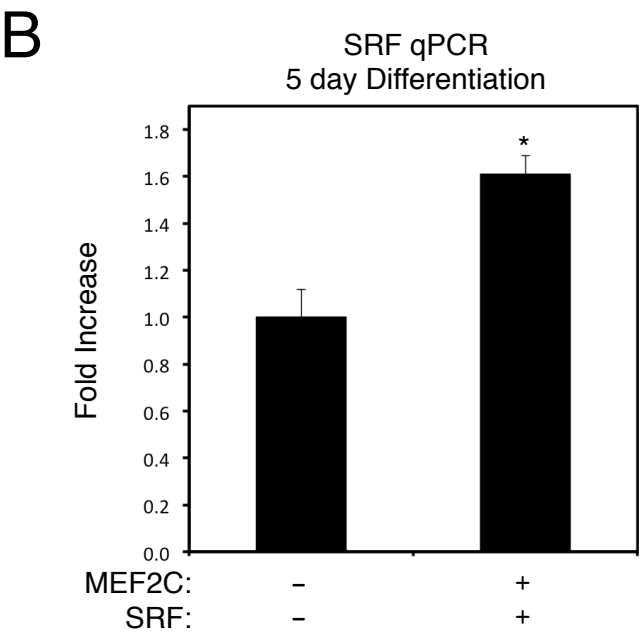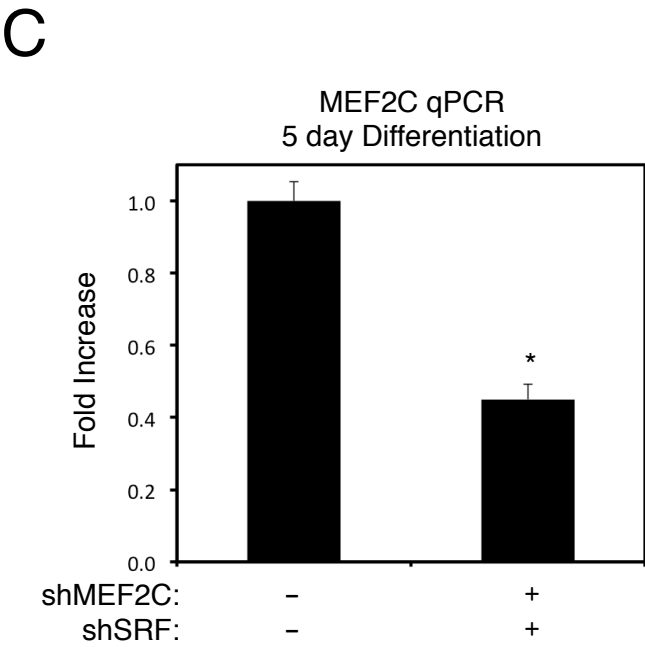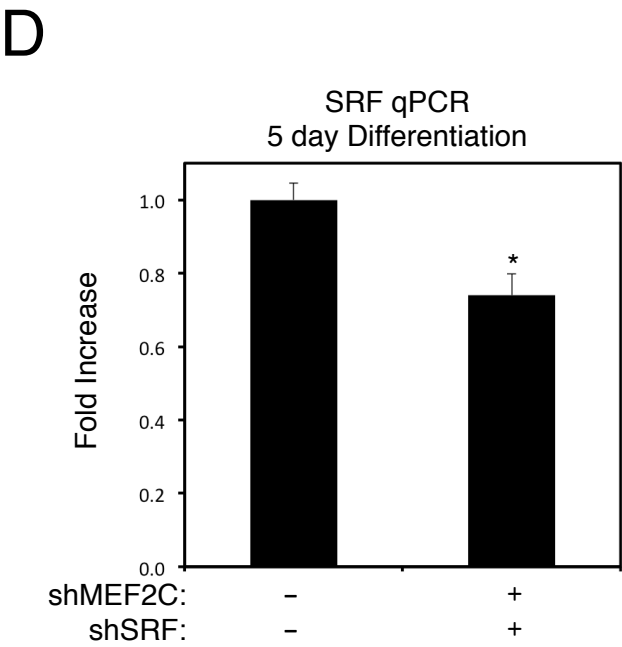

Supplement: Supplementary Figure 3 [file cddis2015306x3.pdf]

Supplemental Figure 3.

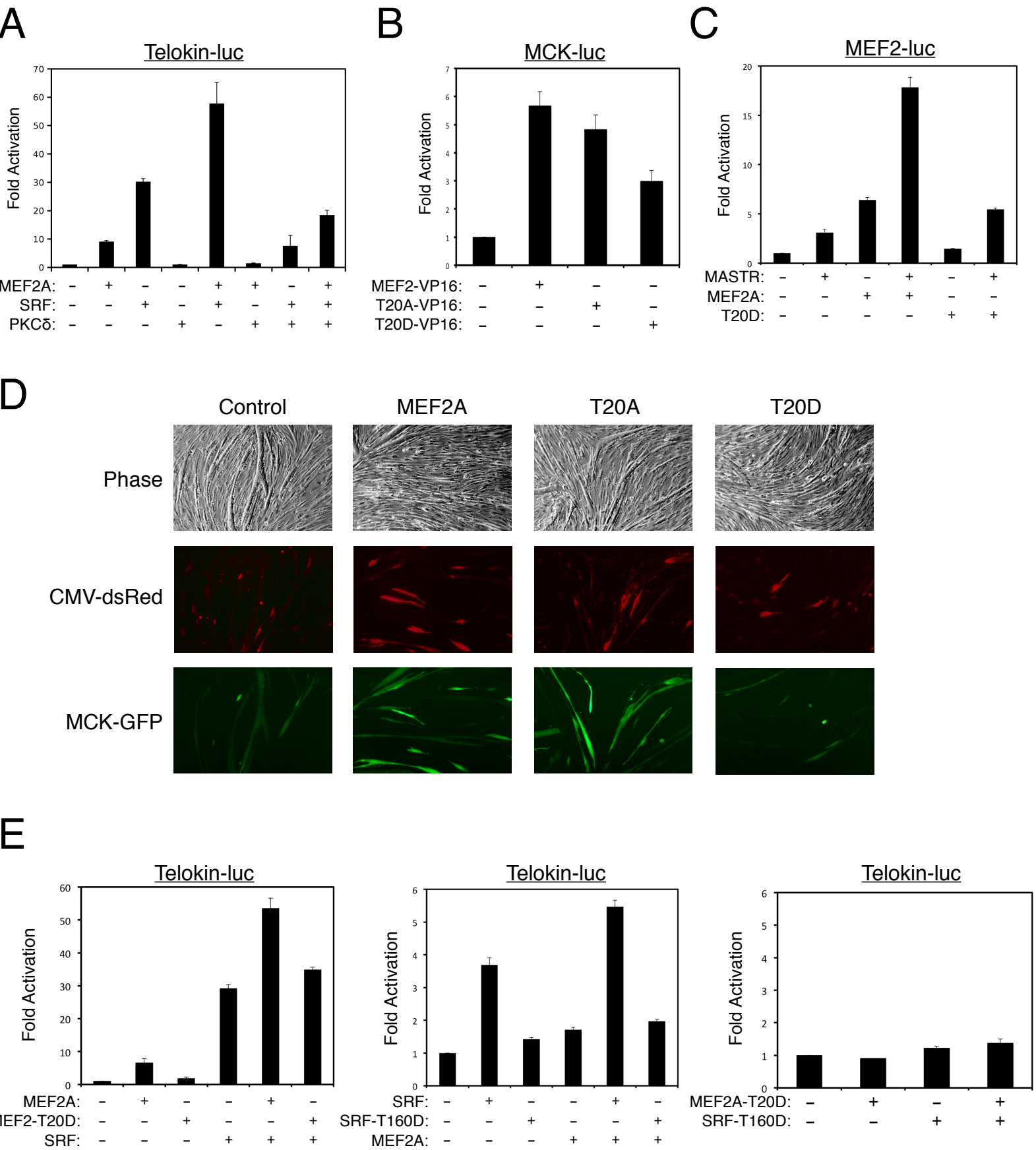

Supplement: Supplementary Figure 4 [file cddis2015306x4.pdf]
