# Supplementary material for: A conserved MADS-box phosphorylation motif regulates differentiation and mitochondrial function in skeletal, cardiac, and smooth muscle cells
Source: Cell Death Dis. 2015 Oct 29;6(10):e1944–. doi: 10.1038/cddis.2015.306 (PMC5399178; doi:10.1038/cddis.2015.306)
Supplement: Supplementary Table 1 [file cddis2015306x5.pdf]

Table 1.

**PPARA (M00242):**	
Aldolase 1, A	NM_007438
Creatine kinase, mitochondrial 2	NM_198415
Ryanodine receptor 2, cardiac	NM_023868
Troponin I, skeletal slow	NM_021467
Calsequestrin 2	NM_009814
Actin-binding Rho activating protein	NM_175456
Syntropin, acidic 1	NM_009228
Myosin-binding protein C, cardiac	NM_008653
Integrin beta 2-like	NM_008405
Myosin, heavy polypeptide II, smooth muscle	NM_013607
**NF-AT (M00302):**	
Aldolase 1, A	NM_007438
Vasoactive intestinal peptide, Vip	NM_011702
Troponin I, skeletal slow	NM_021467
Calsequestrin 2	NM_009814
Hemochromatosis type 2	NM_027126
Actin-binding Rho activating protein	NM_175456
Syntropin, acidic 1	NM_009228
Sodium channel, voltage-gated, type VII alpha	NM_009135
Myosin-binding protein C, cardiac	NM_008653
Integrin beta 2-like	NM_008405
Myosin, heavy polypeptide II, smooth muscle	NM_013607
**STATx (M00223):**	
Aldolase 1, A	NM_007438
Creatine kinase, mitochondrial 2	NM_198415
Troponin I, skeletal slow	NM_021467
Caldesmon 1	NM_145575
Calsequestrin 2	NM_009814
Hemochromatosis type 2	NM_027126
Sodium channel, voltage-gated, type VII alpha	NM_009135
Integrin beta 2-like	NM_008405
**MYF (Wasserman-Fickett PWM):**	
Aldolase 1, A	NM_007438
Ryanodine receptor 2, cardiac	NM_023868
Troponin I, skeletal slow	NM_021467
Calsequestrin 2	NM_009814
Hemochromatosis type 2	NM_027126

Syntropin, acidic 1	NM_009228
Potassium voltage-gated channel Kcnb 1	NM_008420
Sodium channel, voltage-gated, type VII alpha	NM_009135
Myosin-binding protein C, cardiac	NM_008653
**NKX 6.1 (M00424):**	
Ryanodine receptor 2, cardiac	NM_023868
Troponin I, skeletal slow	NM_021467
Hemochromatosis type 2	NM_027126
Actin-binding Rho activating protein	NM_175456
Cholinergic receptor, nicotinic, alpha polypeptide 1	NM_007389
Potassium voltage-gated channel Kcnb 1	NM_008420
Sodium channel, voltage-gated, type VII alpha	NM_009135
Integrin beta 2-like	NM_008405
Myosin, heavy polypeptide II, smooth muscle	NM_013607
**SRF (Wasserman-Fickett PWM):**	
Creatine kinase, mitochondrial 2	NM_198415
Caldesmon 1	NM_145575
Calsequestrin 2	NM_009814
Actin-binding Rho activating protein	NM_175456
Cholinergic receptor, nicotinic, alpha polypeptide 1	NM_007389
Myosin-binding protein C, cardiac	NM_008653
Integrin beta 2-like	NM_008405
Myosin, heavy polypeptide II, smooth muscle	NM_013607
**POU3F2 (M00464):**	
Ryanodine receptor 2, cardiac	NM_023868
Vasoactive intestinal peptide, Vip	NM_011702
Actin-binding Rho activating protein	NM_175456
Cholinergic receptor, nicotinic, alpha polypeptide 1	NM_007389
Potassium voltage-gated channel Kcnb 1	NM_008420
Sodium channel, voltage-gated, type VII alpha	NM_009135
