# Supplementary material for: A conserved MADS-box phosphorylation motif regulates differentiation and mitochondrial function in skeletal, cardiac, and smooth muscle cells
Source: Cell Death Dis. 2015 Oct 29;6(10):e1944–. doi: 10.1038/cddis.2015.306 (PMC5399178; doi:10.1038/cddis.2015.306)
Supplement: Supplementary Table 2 [file cddis2015306x6.pdf]

Table 2.

Identified Diacylglycerols
DG(P-14:0/18:1(9Z))
DG(20:4(5Z,8Z,11Z,14Z)/22:5(7Z,10Z,13Z,16Z,19Z)/0:0)[iso2]
DG(20:4(5Z,8Z,11Z,14Z)/22:5(7Z,10Z,13Z,16Z,19Z)/0:0)[iso2] ESI+175.0V 10.9305
DG(20:2(11Z,14Z)/24:0/0:0)
DG(20:2(11Z,14Z)/24:0/0:0) ESI+175.0V 4.404857
DG(P-14:0/18:1(9Z)) ESI+175.0V 14.152499
DG(18:0/18:0/0:0) ESI+175.0V 14.587999
DG(20:5(5Z,8Z,11Z,14Z,17Z)/22:5(7Z,10Z,13Z,16Z,19Z)/0:0)[iso2] ESI+175.0V 16.466599
DG(20:3(5Z,8Z,11Z)/22:6(4Z,7Z,10Z,13Z,16Z,19Z)/0:0)
DG(20:4(5Z,8Z,11Z,14Z)/22:5(7Z,10Z,13Z,16Z,19Z)/0:0)[iso2] ESI+175.0V 10.931
DG(20:4(5Z,8Z,11Z,14Z)/22:5(7Z,10Z,13Z,16Z,19Z)/0:0)[iso2] ESI+175.0V 10.930333
DG(20:5(5Z,8Z,11Z,14Z,17Z)/22:5(7Z,10Z,13Z,16Z,19Z)/0:0)[iso2] ESI+175.0V 16.456
DG(20:4(5Z,8Z,11Z,14Z)/22:5(7Z,10Z,13Z,16Z,19Z)/0:0)[iso2] ESI+175.0V 14.584
DG(20:5(5Z,8Z,11Z,14Z,17Z)/22:5(7Z,10Z,13Z,16Z,19Z)/0:0)[iso2] ESI+175.0V 16.458334
DG(20:5(5Z,8Z,11Z,14Z,17Z)/22:5(7Z,10Z,13Z,16Z,19Z)/0:0)[iso2] ESI+175.0V 16.459
DG(21:0/22:4(7Z,10Z,13Z,16Z)/0:0)[iso2] ESI+175.0V 3.993333
DG(21:0/22:4(7Z,10Z,13Z,16Z)/0:0)[iso2] ESI+175.0V 3.981
DG(20:1(11Z)/21:0/0:0)[iso2]
DG(O-16:0/18:1(9Z)) ESI+175.0V 4.8475
DG(21:0/22:4(7Z,10Z,13Z,16Z)/0:0)[iso2]
DG(21:0/22:4(7Z,10Z,13Z,16Z)/0:0)[iso2] ESI+175.0V 3.9785
DG(20:2(11Z,14Z)/22:0/0:0)[iso2]
DG(20:5(5Z,8Z,11Z,14Z,17Z)/22:5(7Z,10Z,13Z,16Z,19Z)/0:0)[iso2]
DG(18:0/18:0/0:0)
DG(O-16:0/18:1(9Z))
DG(18:0/18:0/0:0) ESI+175.0V 13.723
DG(20:5(5Z,8Z,11Z,14Z,17Z)/22:5(7Z,10Z,13Z,16Z,19Z)/0:0)[iso2] ESI+175.0V 16.465
DG(20:4(5Z,8Z,11Z,14Z)/22:5(7Z,10Z,13Z,16Z,19Z)/0:0)[iso2] ESI+175.0V 11.043
DG(20:2(11Z,14Z)/24:0/0:0) ESI+175.0V 4.355
DG(P-14:0/18:1(9Z)) ESI+175.0V 12.860001
DG(20:4(5Z,8Z,11Z,14Z)/22:5(7Z,10Z,13Z,16Z,19Z)/0:0)[iso2] ESI+175.0V 10.9305 :15
DG(20:5(5Z,8Z,11Z,14Z,17Z)/22:5(7Z,10Z,13Z,16Z,19Z)/0:0)[iso2] ESI+175.0V 16.46
DG(20:5(5Z,8Z,11Z,14Z,17Z)/22:5(7Z,10Z,13Z,16Z,19Z)/0:0)[iso2] ESI+175.0V 16.458
DG(20:2(11Z,14Z)/24:0/0:0) ESI+175.0V 4.424
DG(22:2(13Z,16Z)/20:1(11Z)/0:0)
DG(20:1(11Z)/22:1(13Z)/0:0)[iso2]
DG(18:0/18:0/0:0) ESI+175.0V 5.053
DG(18:1(11Z)/20:1(11Z)/0:0)
DG(P-14:0/18:1(9Z)) ESI+175.0V 15.0915
DG(18:1(9Z)/19:0/0:0)[iso2]
DG(21:0/22:3(10Z,13Z,16Z)/0:0)[iso2] ESI+175.0V 14.599
DG(20:2(11Z,14Z)/24:0/0:0) ESI+175.0V 4.455
DG(P-14:0/18:1(9Z)) ESI+175.0V 12.841
DG(20:4(5Z,8Z,11Z,14Z)/22:5(7Z,10Z,13Z,16Z,19Z)/0:0)[iso2] ESI+175.0V 11.003
